# The effects of ultrasonic cavitation on the dissolution of lithium disilicate glass

**DOI:** 10.1038/s41598-022-24029-4

**Published:** 2022-11-27

**Authors:** Ben Dillinger, Carlos Suchicital, David Clark

**Affiliations:** Department of Materials Science and Engineering, Virginia Polytechnic Institute and State University, Blacksburg, BA USA

**Keywords:** Characterization and analytical techniques, Scanning electron microscopy, Mass spectrometry, Imaging techniques

## Abstract

There has been little research conducted on how ultrasonic cavitation may affect glass dissolution. The focus of this study was to examine how the mechanisms and kinetics of glass dissolution may change in a system that included ultrasonication. Experiments were conducted on lithium disilicate glass in deionized water at 50 °C between 1 and 7.5 h. Results showed that the erosion from ultrasonication affected the kinetics of glass dissolution. Samples with erosion had 2–3 × more dissolution compared to samples without erosion. The change in dissolution was thought to be partly caused by an increase in the surface area of the sample to volume of solution (SA/V) ratio due to the roughening of the surface and release of particulates and a reduction in the size of the depleted layer due to erosion. Stereoscopic 3D reconstruction of eroded samples was used to calculate the increase in surface area due to erosion. Type 2 surface areas (exfoliation mixed with normal leaching) were roughly 3–6% greater while Type 3 surface areas (heavy roughening of surface) were roughly 29–35% greater than the surfaces areas from Type 1 surfaces (normal leaching).

## Introduction

Ultrasound involves sound waves with frequencies between the 20 kHz and 10 MHz propagated by a transmitter such as an ultrasonic bath wall or homogenizer horn. It is used in a variety of applications including cleaning, dentistry, and enhancing chemical reactions (sonochemistry). In liquid mediums, ultrasound primarily interacts with materials via cavitation. Cavitation creates bubbles that continue to grow until they reach a critical size (related to ultrasonic frequency) before collapsing. Temperatures and pressures in the area of the collapse may reach over 4726 °C and 1000 atm, respectively. A number of physical and chemical effects are generated by the extreme environment caused by bubble collapse. Physical effects include turbulence, shockwaves, agitation, and erosion while chemical effects include sonoluminescence and the generation of radicals (OH^*^ and H* for water). If a bubble is in contact with a solid surface when it collapses it can generate microjets that can impact the material surface at high speed, which contributes to cleaning or eroding the object^1–9^.

Few papers are available that discuss the effects of ultrasound on glass corrosion (chemical dissolution). Virot et al. showed how corrosion changed as function of time for soda-lime silica glasses but did not discuss how ultrasound may change the mechanisms or kinetics of the process^5,7^. These investigators also showed that more erosion (physical removal of material) occurred in harder glasses and observed that corrosion in the soda-lime silica glass increased as a function of power. Papers by Whillock et al. focused on the ultrasonication of 304L stainless steel in nitric acid under various conditions for a two hour time period^8,9^. Whillock et al. found that corrosion had a direct relationship with temperature and an inverse relationship with the distance between the ultrasonic source and the sample. Power and frequency were noted to be interconnected and possibly have optimal settings under which corrosion would peak. Papers by Wei et al.^10^, Tang et al.^11^, and Arnold et al.^12^ examined the dissolution of minerals such as calcite and quartz under sonication. These authors showed an inverse relationship between the hardness/ bond energy of a material and the dissolution enhancement that material saw from sonication. Wei et al. also noted the dissolution enhancement could not be completely explained through the change in surface area caused by erosion or from the formation of radicals for the materials they tested. They proposed that sonication imparts energy onto the solute making it easier for the material to overcome its activation energy for dissolution. The suggested method by which this was accomplished would be “perturbation of the surface atoms from their equilibrium positions.” Tang et al. proposed that, in addition to bond energy, both surface energy and stacking energy play an important role in dissolution enhancement. They suggested that the dissolution enhancement was governed by a dual mechanism through fracture or atomic dislocation caused by sonication.

This work examines the interaction between erosion and corrosion and how ultrasonication affects the dissolution mechanisms and kinetics of a lithium disilicate glass (*Li*_*2*_*O-2SiO*_*2*_). Potential applications for this research may include enhancing the dissolution of rare earth elements from fly ash as was suggested by Tang et al.^11^. There are two main dissolution mechanisms for these types of glasses: ion exchange (Eq. 1) and network dissolution (Eqs. 2 and 3). During ion exchange hydronium ions (*H*_*3*_*O*^+^) diffuse into the glass surface and replace the alkali ions (*R*^+^) bonded to the glass matrix, releasing them into the solution. Byproducts of this reaction include the production of hydroxide ions (*OH*^*−*^) in the solution, the formation of molecular water inside the glass (*H*_*2*_*O (gl)*), and the increase in pH of the system over time (due to the removal of hydronium ions). Network dissolution describes the reaction between the glass matrix and either water or hydroxide molecules which forms silanol bonds (*Si–OH*) around silicon molecules. When a silicon molecule has been completely hydrolyzed it is released into solution as silicic acid. The solubility of silica in solution is very low below pH 9, making this process pH dependent^13–22^.

1 $$\equiv Si-OR\left(gl\right)+{H}_{3}{O}^{+}\left(aq\right)\to \equiv Si-OH\left(gl\right)+{R}^{+}(aq)+{H}_{2}O(gl)$$

2 $$\equiv Si-O-Si\equiv (gl)+{H}_{2}O(aq)\to \equiv Si-OH(gl)+HO-Si\equiv (gl)$$

3 $$\equiv Si-O-Si\equiv (gl)+O{H}^{-}(aq)\to \equiv Si-OH(gl){+}^{-}O-Si(gl)$$

In alkali silicate systems that start below a pH of 9 the rate of ion exchange is initially much faster than network dissolution. The difference in the initial rates leads to the formation and growth of a depleted layer that is relatively devoid of alkali ions. As the depleted layer grows the rate of ion exchange decreases. As the reaction continues the pH of the system will start to increase (due to ion exchange), eventually allowing for more network dissolution to occur. The combination of the decreased rate of ion exchange along with the higher solution pH allows network dissolution to become the main dissolution mechanism by which water interacts with the glass. It is possible to estimate which mechanism is more prevalent at a given point in time by calculating the alpha value for the reaction (Eq. 4). For a binary alkali silicate glass, the equation for the alpha value reduces to the concentration of silica in solution over the concentration of alkali ion in solution. Low alpha values represent systems experiencing primarily ion exchange while higher alpha values represent increasing network dissolution^14,20,23–29^.

4 $$\alpha =\frac{2*[Si{O}_{2}]}{[{R}^{+}]}*\frac{mol\% of alkali oxide \left({R}_{2}O\right)}{mol\% of silica}=\frac{\left[Si{O}_{2}\right]}{\left[{R}^{+}\right]}(for Alkali Disilicate)$$

## Materials and methods

Samples were made using a lithium disilicate glass frit manufactured by Specialty Glass, Inc. (Oldsmar, FL, USA). The composition of the material was verified by Spectrochemical Laboratories—Materials Evaluation, INC using an inductively coupled plasma technique (ICP). This information is included in Supplementary Table S1 and includes the expected wt. % for each individual component in addition to the ICP results. Monolithic samples of glass were produced by melting and casting the frit into rods (12.7 × 12.7 × 152 mm) using a graphite mold. After solidification the resulting rod was annealed overnight at 400 °C. Samples with an approximately 4.5 mm thickness were cut from the annealed rod and the cut faces were ground with silicon carbide paper using up to a grit of 600 and washed using isopropyl alcohol (IPA). The average surface area of the ground samples was ~ 4.6 cm^2^. Microscopy of the ground, unleached surfaces showed scratch marks and microcracks. Dissolution experiments used deionized (DI) water with a measured resistance between 16.7 and 17.5 MΩ.

**Figure 1:**
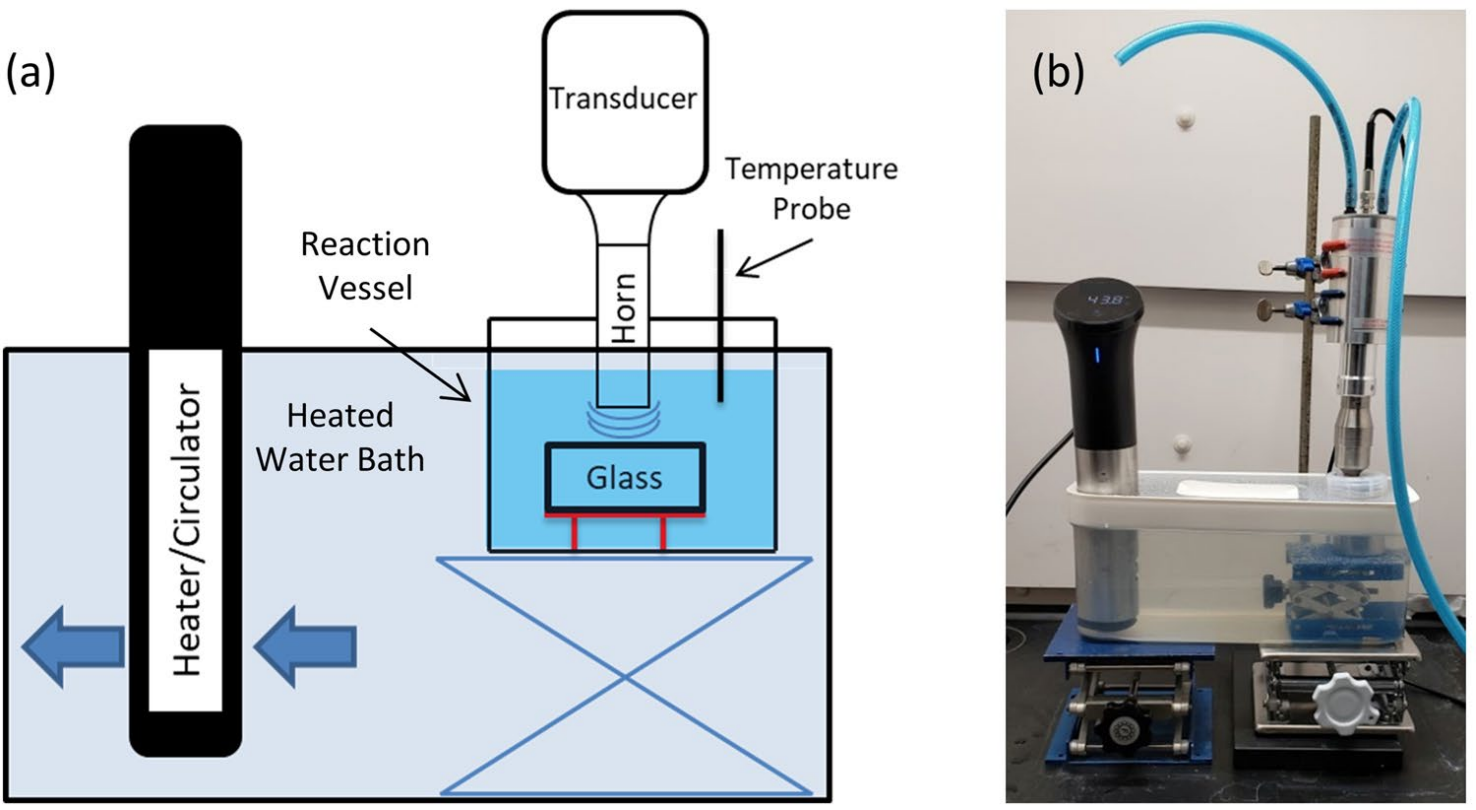
Schematic created using PowerPoint (**a**) and apparatus (**b**) used for ultrasonic dissolution experiments. Experiments took place in a stainless steel reaction vessel which had the reaction temperature was regulated by a surrounding water bath.

Two sets of glass dissolution experiments were conducted for this research. The first were static experiments used as a baseline for comparison while the second included ultrasonication as an additional stimulus. Ultrasonic experiments used a Qsonica Q500 Ultrasonic Homogenizer with a frequency of 20 kHz. The homogenizer’s horn was made from Ti-6Al-4V alloy and was resistant to the higher pH of the reacted solution. To prevent the transducer from overheating, filtered, compressed air was used as a coolant at 10–20 psi. Static experiments used a 90 mL polyfluoroalkoxy (PFA) container while ultrasonic experiments used a 100 mL 316 stainless steel container as the reaction vessel. The stainless steel was selected as its higher thermal conductivity allowed for better control of the reaction temperature and the corrosion resistance of the steel meant that it should have minimal interaction with the dissolution system. To minimize heating effects caused by ultrasonication, the reaction vessel was placed in a temperature-controlled water bath that was set 4–6 °C lower than the reaction temperature using an Anova heater/circulator (Range: 0–92 °C) (Fig. 1). The lid of the reaction vessel protruded through the lid of the water bath container and had holes for the homogenizer horn and a thermocouple. Glass samples were placed inside the reaction vessel on a polytetrafluoroethylene (PTFE) stand with a polyether ether ketone (PEEK) mesh bottom used in both sets of experiments. Type K thermocouples were used to monitor the solution’s temperature. For this study the reaction temperature was set to 50 °C and the experiment duration varied between 1, 4, or 7.5 h. A new experiment was conducted for each experiment duration. The power intensity (output power divided by the horn area) for ultrasonic experiments was 10 or 20 W cm^−2^. All experiments started with a surface area of the sample to volume of solution (SA/V) ratio of 0.1 cm^−1^, though this was shown to change over time for samples that experienced erosion.

Once the DI water was at temperature the sample was added to the reaction container. Next the homogenizer horn was activated for ultrasonic experiments or the reaction vessel was placed into a LabLine Imperial V convection oven and an overhead stirrer was activated for static experiments. To place the horn at the same depth in the reaction vessel each time, a positioning line was added to the horn based on measurements of the apparatus. The distance from the sample surface to the horn tip was nominally 2 mm but could vary slightly with sample thickness. A slight gap was left between the lid and the overhead stirrer or the homogenizer horn to prevent damage from occurring to the equipment while it ran. At the end of an experiment the samples were removed and the solutions were weighed to correct for evaporation and an aliquot saved for elemental analysis.

Solutions from the 4 and 7.5 h ultrasonic experiments showed large amounts of dark, metallic particulates. Minimal amounts of these particulates were seen after 1 h of sonication. Qsonica indicated that these were likely titanium alloy particulates and should be expected with normal use of the instrument. These particulates were filtered with 0.4 µm filter paper before solutions were chemically analyzed. Energy dispersive x-ray spectroscopy (EDS) of the washed particulates was conducted to see if the titanium could induce silicate precipitation in the same manner to iron creating silicates from solution^30,31^. Due to the limitations of EDS only silicon and not lithium could be checked with this method. Results (Supplementary Fig. S1) showed the elements of the sonicator horn along with the iridium the particles were coated with. Carbon and oxygen were also present and are likely from exposure to the atmosphere and the carbon tape that the particles were attached to. Silicon was not found at a level above the minimum detection limits for the EDS.

Solutions were analyzed using inductively coupled plasma mass spectroscopy (ICP-MS) to measure the amount of silicon and lithium that had been dissolved (Thermo Electron X-Series ICP-MS instrument). Before analysis solutions were acidified by adding 2 vol.% of nitric acid and 1 vol. % of hydrochloric acid.

Results include data for the normalized mass loss (g cm^−2^) and alpha value versus time along with microscopy and stereoscopic reconstructions of the reacted surfaces. Normalized mass loss (*NL*) plots were calculated using Eq. (5).

5 $$NL=\frac{{C}_{i}}{{M}_{i}}*\frac{V}{SA}$$

In Eq. (5), *C*_*i*_ is the measured concentration (ppm) of an element, *i*, in solution, *M*_*i*_ is the mass fraction of that element in the glass sample, *V* is the volume of solution (L) which has been corrected for evaporation, and *SA* is the surface area of the sample (cm^2^)^30,32–37^.

A JEOL IT500 SEM at 5 kV with a secondary electron detector was used to observe the sample surfaces. Prior to analysis the samples were coated with 5 nm of iridium using a Leica ACE600 or a Cressington 208HR with silver paint applied to the edges to increase specimen conductivity. Stereoscopic 3D reconstruction was conducted using the Digital Surf MountainsSEM program. This program has been used to analyze the surface morphology of many materials^38–42^ and was suggested over AFM based on the estimated feature size of the eroded regions.

Stereoscopic 3D reconstructions were performed on multiple sections of each type of surface to quantify the changes in surface behavior. Analysis of the reconstructions includes a 3D view of the surface, a depth profile for some regions, and a table (Supplementary Tables S2-S4) comparing the changes in surface area. A ratio of the developed surface area from the reconstruction (surface area calculation that takes into account the 3D features of the image) to the projected horizontal area of the micrograph (SA/PA ratio) was calculated to determine the effects of erosion along with a percent increase in the surface area. The results of these analyses include the mean surface area change and tables with the individual values are included as Supplementary Information. With the exception of the normal dissolution surfaces (the software had issues trying to level these relatively flat surfaces), the 3D views for the reconstructions were leveled and all reconstructions had their features normalized. Leveling aligns a slanted surface without changing the area calculations so the comparison of normal surfaces to eroded surfaces is still possible.

## Results and discussion

### Surface microscopy and reconstruction

**Figure 2:**
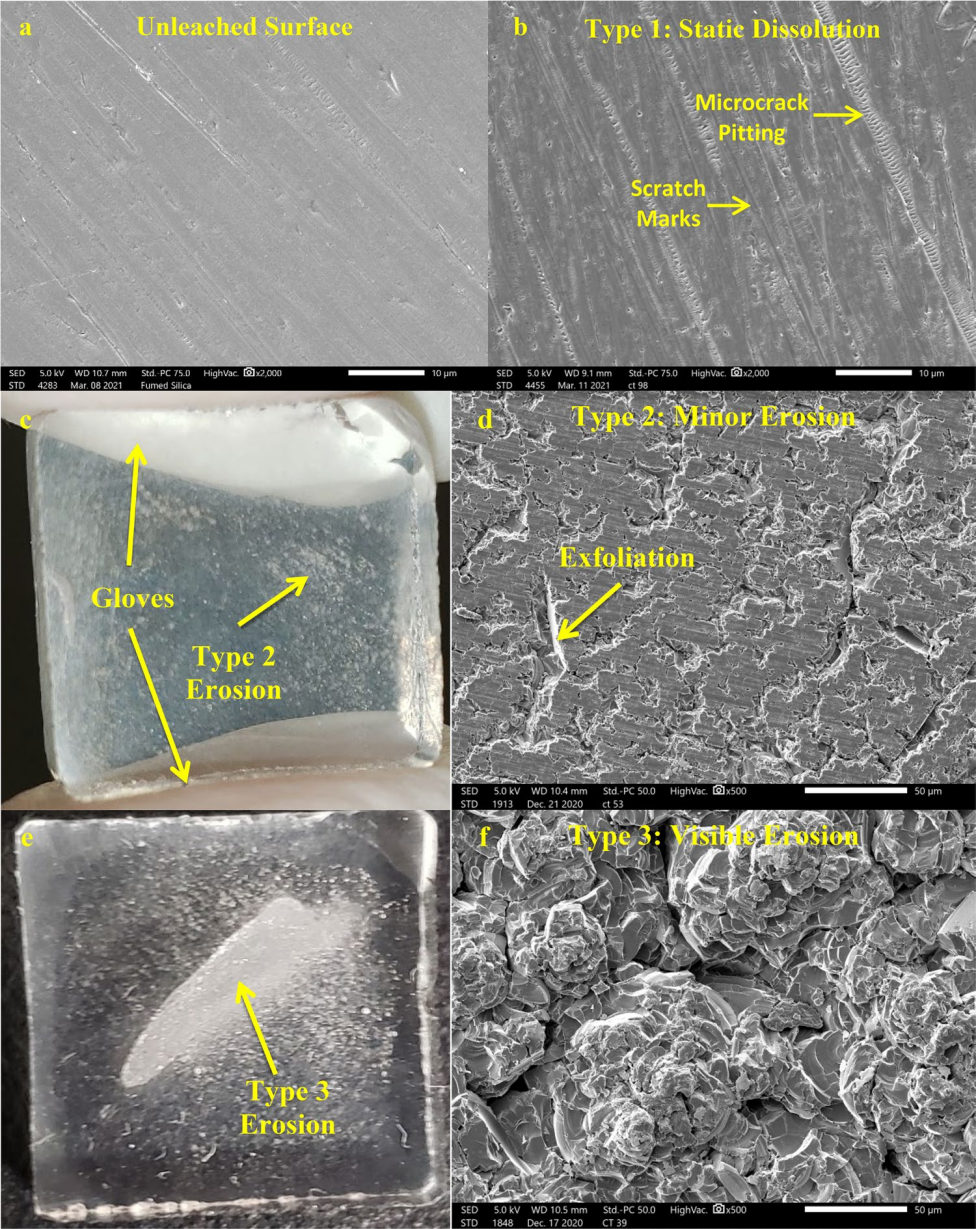
Microscopy of unleached and leached surfaces. Unleached glass surface (**a**) at 2000 × showing scratch marks and light microcrack pitting present. Type 1/static dissolution surface at 2000 × (**b**) using a micrograph from a 7.5 h experiment. These regions show preferential dissolution of scratch marks and microcracks. Type 2 surface features at a macroscopic (**c**) and 500 × (**d**) view taken from a from a 20 W cm^−2^, 7.5 h experiment. Surface characterized by exfoliation of the glass surface around areas of leached scratch marks. Type 3 surface features at a macroscopic (**e**) and 500 × (**f**) view taken from a 10 W cm^−2^, 1 h experiment. Surfaces are characterized by heavy erosion that has completely altered the morphology resulting in significant roughening. The glass is visibly opaque in these regions.

**Figure 3:**
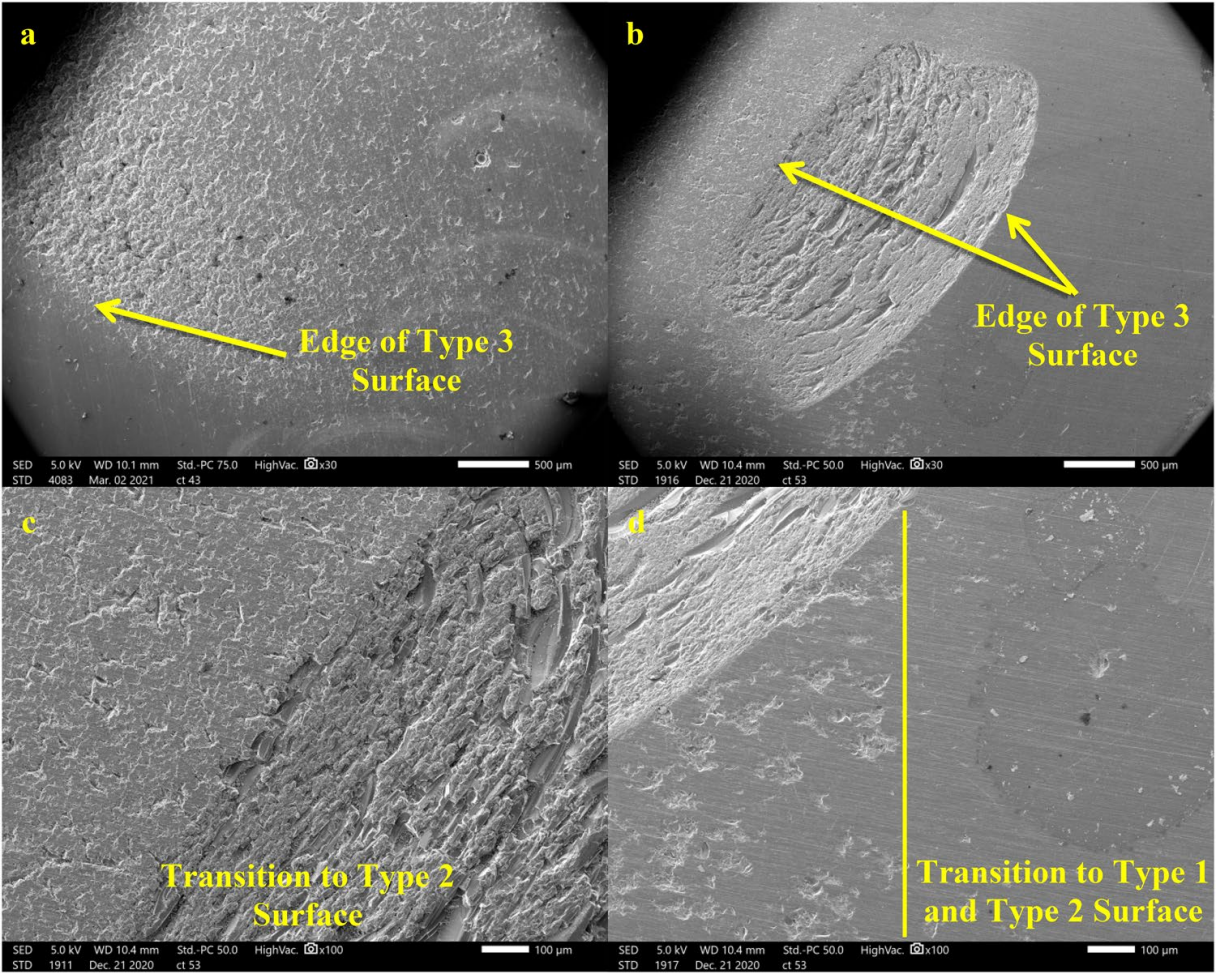
Edge of Type 3 erosion at 30 × (**a**,**b**) and 100 × (**c**,**d**) magnification. c shows a transition to a Type 2 surface while d shows a transition to both Type 1 and Type 2 surfaces with a yellow line added to show the separation between the regions. The micrograph in a was from a 20 W cm^−2^, 1 h experiment while the micrographs for b-d are from a 20 W cm^−2^, 7.5 h experiment.

Microscopy of ultrasonicated samples have been divided into three types of surfaces for this research. Each of these surface types may appear on ultrasonicated samples and do no appear to correlate to the experimental conditions. Preliminary microscopy of the unleached glass surface is also included in Fig. 2a. This surface has some scratch marks and the beginnings of microcrack pitting. Type 1 surfaces (Fig. 2b) are regions with little to no erosion from ultrasonication or are from static experiments. They display normal dissolution features where the preferential dissolution of scratch marks and microcracks has occurred. Type 2 surfaces (Fig. 2c,d) are characterized by regions where large chunks of material have been exfoliated due to ultrasonication. These exfoliation features are visible in normal light if a large number of them are clustered together (Fig. 2c). Normal dissolution features are visible in between the exfoliated surfaces. Type 3 surfaces (Fig. 2e,f) show extreme erosion of the glass to the point where these regions are visibly opaque. These regions have a rough texture with large variations in height that is unique compared to other surfaces. The size of Type 3 surface features should vary based on both the distance between the horn^7,9^ (due to variations in the sample thickness after grinding) and the sample and their parallel alignment. Type 3 surfaces have a distinct edge where they transition into a Type 1 or Type 2 region (Fig. 3). Type 2 and Type 3 surfaces only appear on the surface that was directly facing the homogenizer horn.

**Figure 4:**
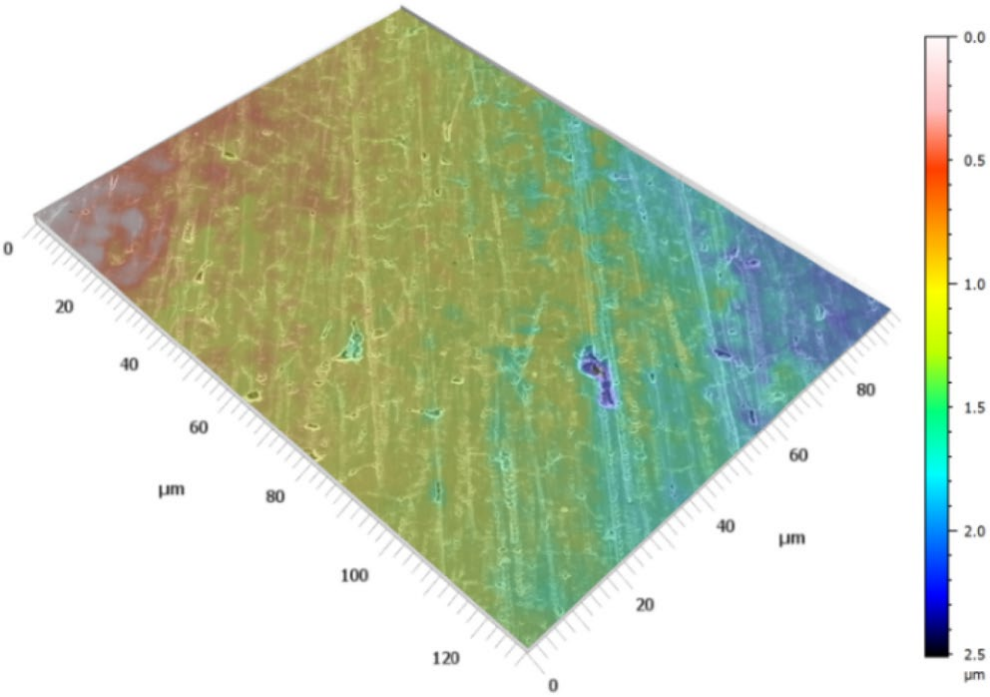
3D reconstruction of a Type 1/Normal leaching Surface from a 10 W cm^−2^, 7.5 h experiment created using MountainsSEM. Minimal to no erosion has occurred. Changes in surface area are caused by preferential leaching of scratch marks and microcracks. The leveling function of MountainsSEM has trouble working on relatively flat surfaces so the slight downward slope of the reconstructed surface has not been corrected.

A reconstruction for normal dissolution surfaces (Type 1) is shown in Fig. 4. Outside of a few larger pits, the scratch marks and other dissolution features are shallow and barely affect the reconstruction at this scale. Data from the reconstructions shows that the mean change in surface area is 0.3% (Supplementary Table S2). This value is the change in surface area caused by normal leaching of the glass surface and should be used when analyzing the other types of surfaces.

**Figure 5:**
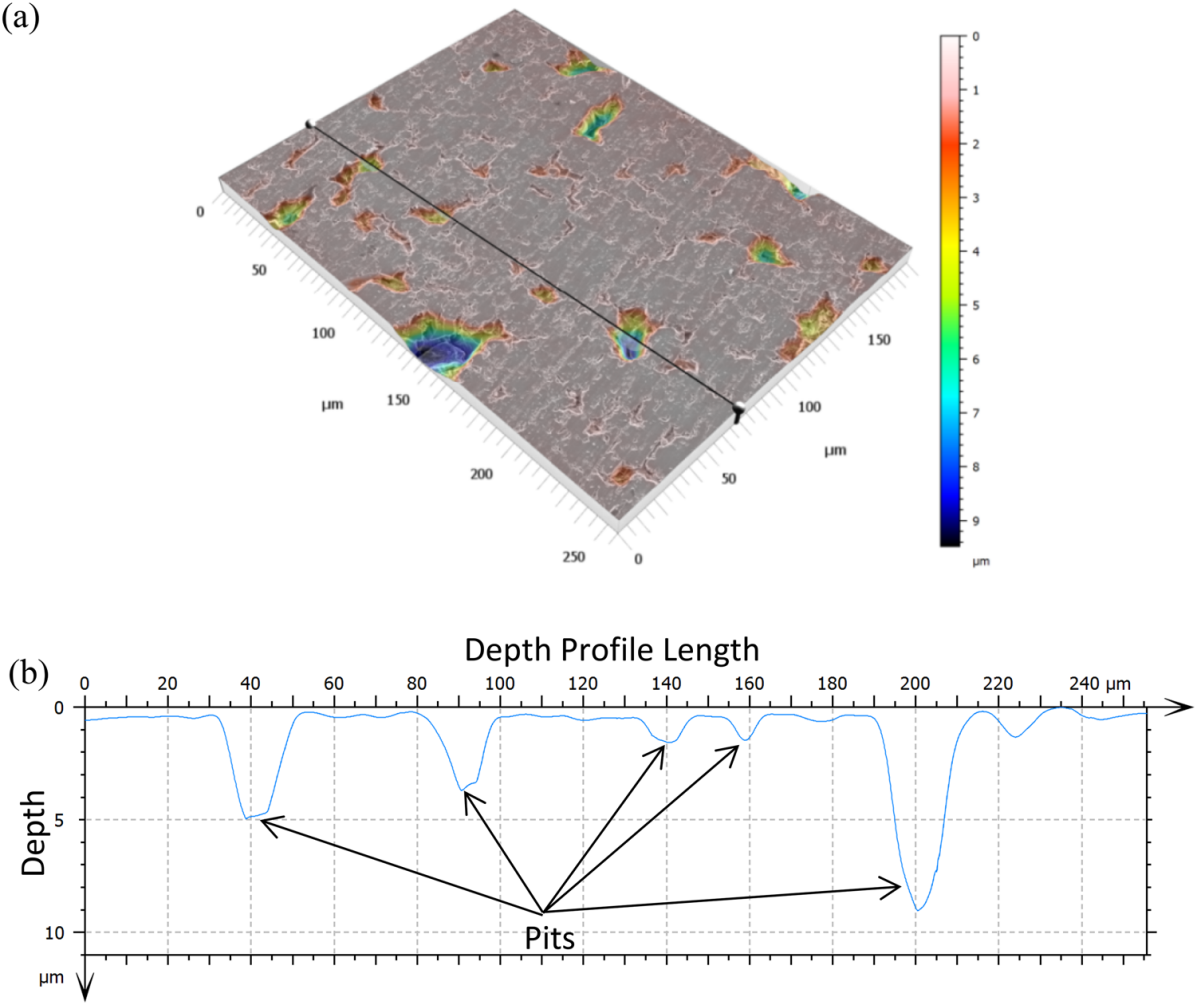
3D reconstruction of a Type 2 surface from a 20 W cm^−2^, 1 h experiment created using MountainsSEM (**a**). The line across the image designates where the depth profile (**b**) was taken. The additional pitting from erosion has caused the surface area to slightly increase compared to a surface with normal leaching features. Pits appear to be between 1 and 10 microns in depth.

Figure 5a presents a reconstruction for a Type 2 surface. The line drawn across the image represents where the depth profile (Fig. 5b) was taken. The depth profile shows a relatively flat surface dotted with pitting ranging from 1-9 µm in depth. Based on the profile the small, shallow pits are roughly 1–2 µm deep, the medium pits are about 5–7 µm deep, and the largest pits reach up to 9 µm deep. Supplementary Table S3 displays the surface area analysis of Type 2 surfaces from multiple samples. The SA/PA ratio for these regions shows a slight increase in surface area due to erosion compared to Type 1 surfaces (0.3% for Type 1 compared to 3–6% for Type 2). There is little difference due to power intensity but the surface area does show a slight increase due to time with erosion going from ~ 3% after 1 h to ~ 6% after 7.5 h.

**Figure 6:**
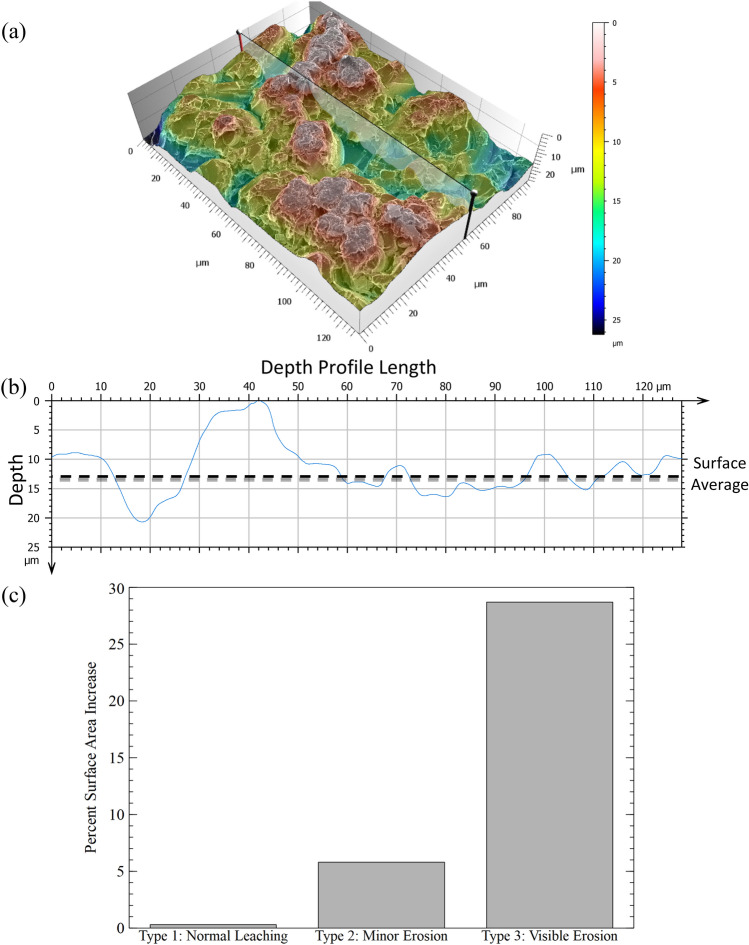
3D Reconstruction of a Type 3 surface from a 10 W cm^−2^, 1 h experiment created using MountainsSEM (**a**) showing the heavy roughening of the glass surface due to ultrasonication. This type of surface is visible macroscopically. The line across the image indicates where the depth profile (**b**) was taken. The average surface height is marked with a dashed line in the depth profile. The largest surface features are ~ 10–15 microns different from the average. The bar chart made with Veusz (**c**) compares the surface area changes measured from the reconstructions for each type of surface. The results for Type 2 and 3 Surfaces come from 20 W cm^−2^ experiments.

**Figure 7:**
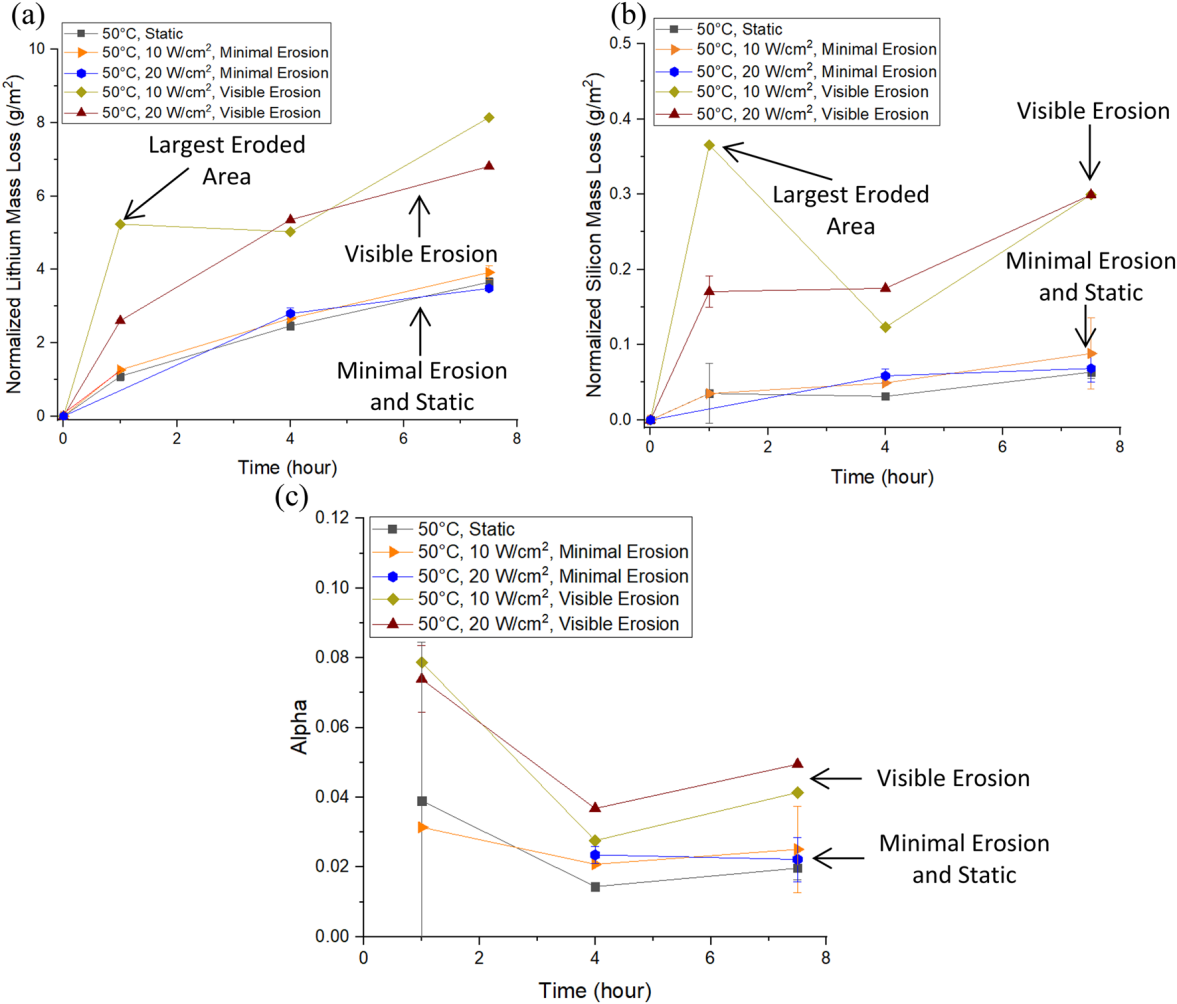
Lithium normalized mass loss (**a**), silicon normalized mass loss (**b**), and alpha value (**c**) plots for ultrasonic experiments created using OriginPro. Static experiments are included for comparison. Results show that samples with any amount of visible erosion have a ~ 2–3 × increase in dissolution compared to samples from other experiments. The experiment with the sample that produced the largest Type 3 surface also has a much higher amount of dissolution compared to other data points from the same time frame. This could indicate the potential change in dissolution due to cavitation under optimal conditions.

Figure 6a illustrates a reconstruction for Type 3 surfaces along with the line for the depth profile (Fig. 6b). Unlike the relatively flat surfaces with scattered pitting that characterized Type 2 surfaces, Type 3 surfaces show large variations in the height or depth of their features due to roughening. The tallest peaks and deepest pits are roughly 10–15 µm above or below the surface average (dashed line on the depth profile). Supplementary Table S4 shows the results of the surface area analysis for Type 3 surfaces. Results from 1 and 7.5 h experiments at both power intensities are included in the table. With the exception of the 20 W cm^−2^, 1 h experiments, only one sample showed visible erosion for each experimental condition and thus the different projects listed in the Tables represent different locations across the same sample. Based on the results there is little change in the surface area due to time or power intensity with the exception of the 7.5 h, 10 W cm^−2^ data. Type 3 surfaces had a > 10% change in surface area compared to Type 1 surfaces. Most reconstructions showed that Type 3 surfaces have a 29–35% increase in surface area while the sample from 7.5 h, 10 W cm^−2^ experiment showed a 13% increase. Normalized mass loss results (Fig. 7) are similar between the 1 and 7.5 h experiments at 10 W cm^−2^ even though the eroded surface area of the latter was smaller. Figure 6c provides a visual aid for the surface area changes. Results for the Type 2 and 3 surfaces come from the 20 W cm^−2^ reconstructions.

### Dissolution results

The dissolution results for sonicated samples were divided into two categories based on the surface features in addition to the distinctions for power intensity. Dissolution results with the label “Visible Erosion” are from samples that have any amount of Type 3 surfaces. Results with the label “Minimal Erosion” are from samples with mostly Type 1 surfaces mixed with scattered erosion from Type 2 regions. Figure 7a,b shows the lithium and silicon NL for ultrasonic experiments along with static experiments for comparison. Figure 7c shows the alpha value for all experiments. Data points where an experiment was repeated have error bars attached to them that represent the standard deviation from the two experiments with the data point itself representing the mean value of the experiments. Results without the error bar represent only one experiment. The sample with the largest amount of erosion (10 W cm^−2^, 1 h experiment that formed a circle of similar size to the sonicator horn similar to what Virot et al. presented^7^) shows the potential change in dissolution under optimal erosion conditions. Samples with “Minimal Erosion” have nearly identical dissolution results to static experiments while those with “Visible Erosion” show a noticeably higher amount of dissolution (2–3 × higher for most data points). Visibly eroded samples also have slightly higher alpha values compared to static and minimal erosion experiments. This indicates that these experiments experienced slightly more network dissolution than their counterparts though for the timeframe used were still primarily reacting via ion exchange. pH measurements for systems with visible erosion were similar to static dissolution experiments but were ~ 0.5–1.0 higher than minimal erosion systems. This may indicate that more ion exchange occurred during ultrasonic experiments with any amount of visible erosion compared to those with minimal erosion. It is unclear why the static experiments had similar pH values to visible erosion experiments while their normalized dissolution was similar to minimal erosion experiments.

Dissolution results combined with the microscopy analysis indicates that erosion affects the kinetics of glass dissolution. Changes in morphology have been associated with enhanced sonochemistry for other material systems^6,10–12^. Erosion roughens the glass surface and releases particulates into solution causing the SA/V ratio of the reaction system to increase due to an increase in surface area. The higher SA/V ratio allows the dissolution reactions to proceed at a greater apparent rate.

**Figure 8:**
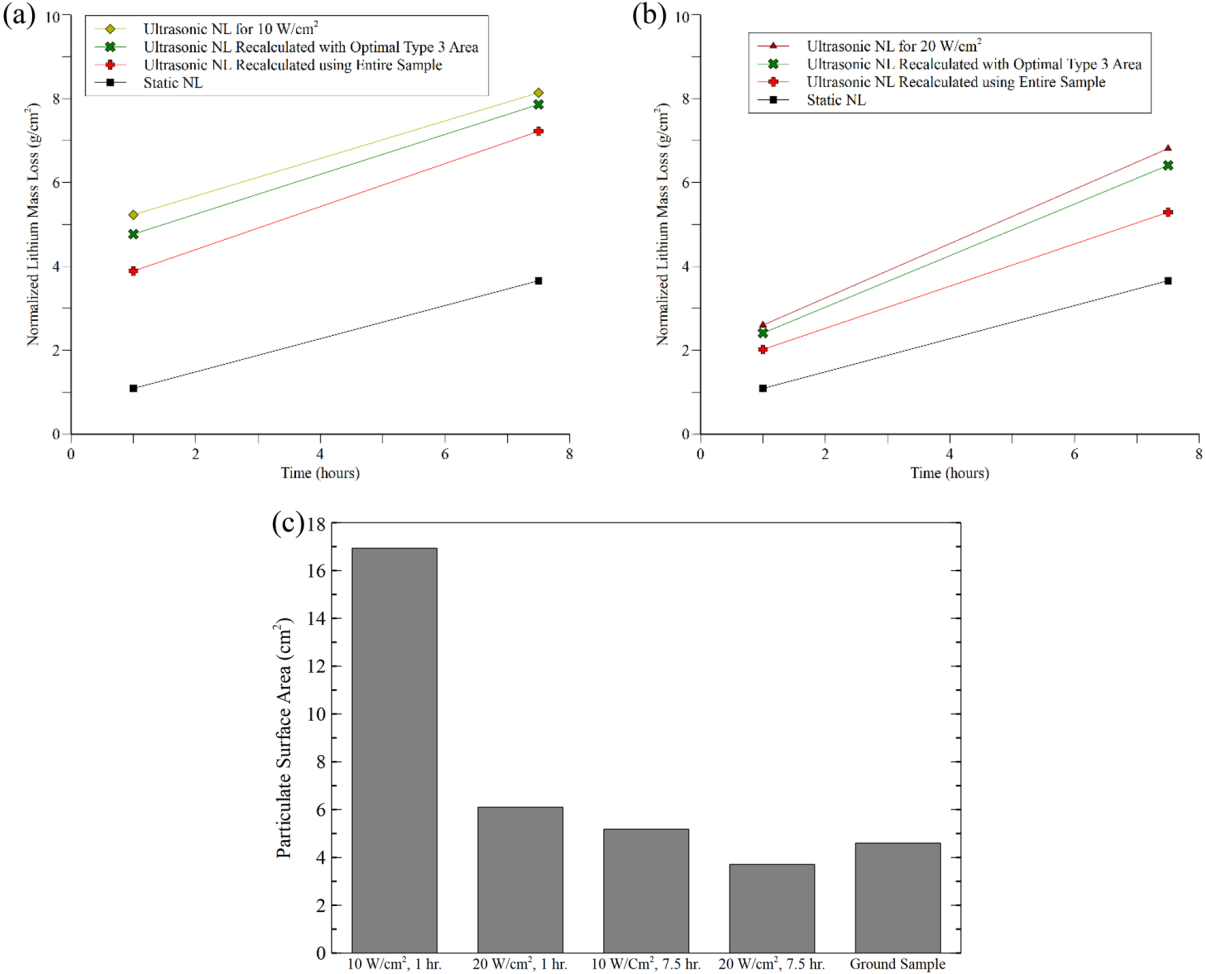
Original and recalculated normalized lithium mass loss for 10 W cm^−2^ (**a**) and 20 W cm^−2^ (**b**) results using Veusz. Recalculations were completed assuming a sample experienced Type 3 erosion in the shape of the homogenizer horn (optimal conditions) or with the extreme assumption of the entire sample experiencing Type 3 erosion. Even in the extreme case where the entire sample experienced erosion, the recalculated normalized mass loss is still higher than static dissolution results. The additional particulate surface area required to make the ultrasonic results equivalent to static results is shown in the bar chart (**c**). The average surface area of a ground, uncorroded sample is included in the chart for comparison. For most results the particulates would need to create additional surface area equivalent to a second unleached sample.

As noted by Wei et al., Tang et al., and Arnold et al. dissolution enhancement cannot be completely explained by the change in surface area from erosion. This can be seen in Fig. 8 where the lithium dissolution results for 10 W cm^−2^ (a) and 20 W cm^−2^ (b) “Visible Erosion” experiments have been renormalized at 1 and 7.5 h to account the change in surface area measured from the reconstructions. Renormalization has been conducted using the simplification that the Type 3 erosion occurred under optimal conditions where it had a similar size to the homogenizer horn and that the change in the surface area from Type 2 surfaces would be negligible. While this is not true for all samples, it will provide a baseline for the analysis. For an extreme example, the data has also been renormalized using the assumption that the entire glass surface had experienced Type 3 erosion. Renormalized values for optimal conditions only show a ~ 3–9% decrease in the dissolution. Even in the extreme case where the entire surface experienced Type 3 erosion there is only a ~ 12–25% drop in dissolution. When compared to static dissolution experiments the more realistic normalization values were still ~ 1.7–2.2 × higher for most data points with the 10 W cm^−2^, 1 h system being over 4 × higher. Figure 8b presents the additional surface area that particulates would need to make for the ultrasonic normalized mass losses to be equivalent to their static counterparts along with a comparison value from ground, uncorroded samples. For most results the additional surface area would be roughly equal to adding a second uncorroded glass sample to the system, with the 10 W cm^−2^, 1 h system requiring the equivalent of four additional uncorroded samples. Based on these observations it seems unlikely that the dissolution enhancement for lithium disilicate glass can be completely explained by a change in surface area. This aligns with the observations by Wei et al., Tang et al., and Arnold et al. for the dissolution of other materials.

An additional mechanism to consider would be the effect erosion has on the depleted layer. Erosion of the surface releases glass into solution, which would cause the size of the depleted layer to shrink at a faster rate than would be possible under normal dissolution conditions. A smaller depleted layer would allow more ion exchange to occur, which could cause the solution to transition from a relatively neutral pH to a basic pH faster than under normal conditions. The higher solution pH would allow the network dissolution rate to increase at an earlier point in the reaction. This could explain why the visible erosion experiments showed slightly higher alpha values compared to their minimal erosion counterparts.

The amount of dissolution was relatively consistent between the different power intensities tested. This was surprising as Virot et al. showed dissolution increased with the power intensity for their soda lime silicate glass which has similar mechanisms for dissolution. One explanation could be that the size variation of the eroded area (see observations for Type 3 surfaces) could be obscuring potential effects related to power intensity.

## Conclusion

Ultrasonication produced multiple types of erosion surfaces on lithium disilicate glass. These include surfaces with minimal to no erosion and normal dissolution features (Type 1), surfaces with exfoliation mixed with normal dissolution features (Type 2), and surfaces that have experienced heavy roughening from erosion (Type 3). Both the distance between a sample and the homogenizer horn and their alignment appeared to impact the amount of Type 3 erosion that occurs. The experiments where minimal erosion occurred had similar dissolution compared to static experiments with no ultrasonication while experiments with Type 3 surfaces showed a 2–3 × increase in dissolution for both elements under most experimental conditions. These results suggested that erosion changes the kinetics of glass dissolution. Erosion causes the SA/V ratio for the system to increase by both roughening the glass surface (roughly a 3–6% increase for Type 2 and roughly a 29–35% increase for Type 3) and releasing small particulates into solution. Increasing the surface area alone did not fully explain the increase in dissolution. When dissolution was renormalized to take into account the measured change in surface area due to erosion it was still greater than the dissolution measured for static experiments. It seems unlikely that the formation of particulates would account for the surface area required to make dissolution from sonication equivalent to static results. Particulates would need to create additional surface area roughly equivalent to that of an additional uncorroded sample. This aligns with the observations of Wei et al., Tang et al., and Arnold et al. that an additional mechanism contributes to dissolution enhancement. Those authors theorized that this mechanism was sonication providing energy to the solute to make it easier for it to overcome the activation energy for dissolution. An additional effect to consider is the shrinking of the depleted layer due to erosion, which should occur at a faster rate than would be possible under from network dissolution under static conditions. This should prevent the rate of ion exchange from decreasing in the affected region, which in turn may cause the pH of the system to increase at a faster rate than expected. The increase in pH could allow the rate of network dissolution to increase earlier, leading to the slightly higher alpha values observed. Future work for this research should attempt to measure dissolution from samples that experience a consistent amount of Type 3 Erosion. This would confirm if there is a change in the dissolution from power intensity that had been obfuscated variations in the size of the eroded area. Another area of importance would be measuring the number of particulates created through erosion and determining if the dissolution of lithium disilicate is also unaffected by the formation of radicals similarly to what was reported by Wei et al.

## Supplementary Information

Supplementary Information.

## Data Availability

The raw/processed data required to reproduce these findings may be found by emailing the corresponding author.
