# Supplementary material for: The effects of ultrasonic cavitation on the dissolution of lithium disilicate glass
Source: Sci Rep. 2022 Nov 27;12:20398. doi: 10.1038/s41598-022-24029-4 (PMC9701792; doi:10.1038/s41598-022-24029-4)
Supplement: Supplementary file 1 — Supplementary Information. [file 41598_2022_24029_MOESM1_ESM.pdf]

Supplementary Material

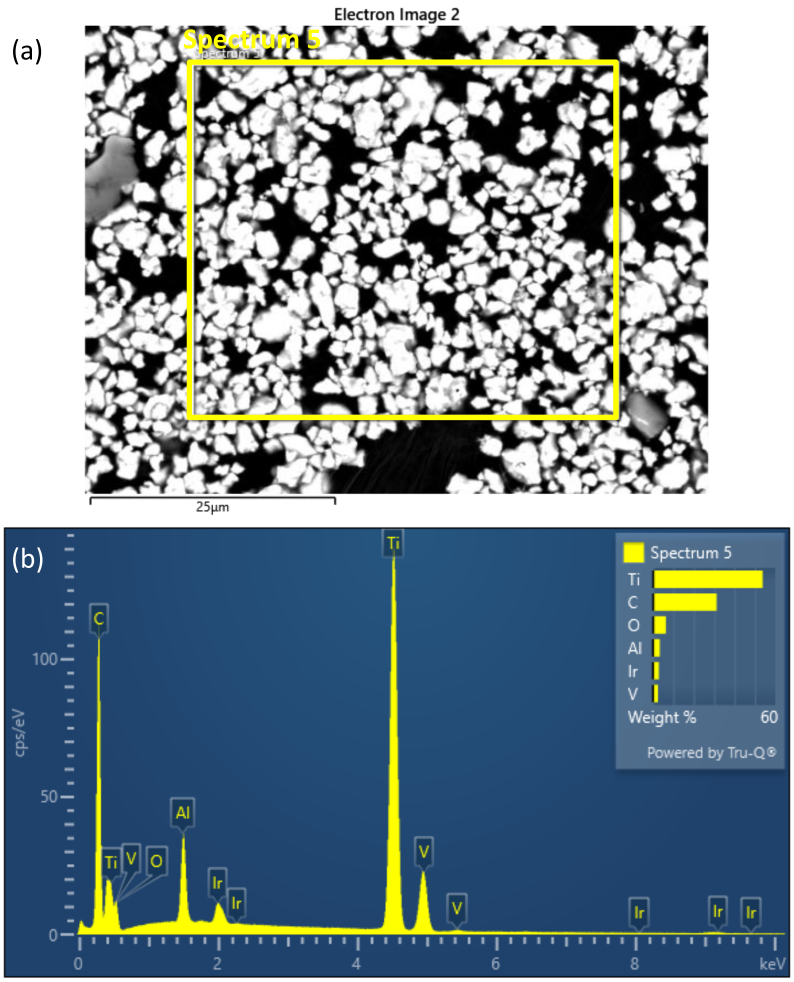

Supplementary Figure 1. Microscopy of the titanium particulates where EDS was conducted (a) and the EDS spectrum of the area (b). EDS shows the elements of the sonicator horn (titanium, vanadium, and aluminum) along with the iridium used to coat the particles. Carbon and oxygen are also shown and are likely from the carbon tape and the atmosphere. No silicon is present which indicates that it was not leached from solution.

Supplementary Table 1: Expected Composition for Lithium Disilicate Glass and ICP Results from Spectrochemical Laboratories

Material	Expected Composition (wt. %)	Spectrochemical Laboratories Analysis (wt. %)
Li_2O	19.91	17.88
SiO_2	80.09	81.03

Supplementary Table 2: Surface Area Measurements for Type 1/Normal Leaching Surfaces

Type 1 Surfaces	SA/PA ratio	Mean Ratio	Mean Percent SA Increase
Projection 1	1.003	1.003	0.3%
Projection 2	1.002		

Supplementary Table 3: Surface Area Measurements for Type 2 Surfaces

Dissolution Conditions		SA/PA ratio	Percent SA Increase
7.5 hr., 10 W cm^{-2} Reconstructions	Projection 1	1.061	6.1%
	Projection 2	1.034	3.4%
Mean		1.048	4.8%
1 hr., 20 W cm^{-2} Reconstructions	Projection 1	1.024	2.4%
	Projection 2	1.036	3.6%
	Projection 3	1.023	2.3%
Mean		1.028	2.8%
7.5 hr., 20 W cm^{-2}	Projection 1	1.058	5.8%

Supplementary Table 4: Surface Area Measurements for Type 3 Surfaces

Dissolution conditions		SA/PA Ratio	Percent SA Increase
1 hr., 10 W cm^{-2} Reconstructions	Projection 1	1.308	30.8%
	Projection 2	1.333	33.3%
	Projection 3	1.406	40.6%
Mean		1.349	34.9%
7.5 hr., 10 W cm^{-2} Reconstructions	Projection 1	1.109	10.9%
	Projection 2	1.120	12.0%
	Projection 3	1.152	15.2%
Mean		1.127	12.7%
1 hr., 20 W cm^{-2} Reconstructions	Projection 1	1.317	31.7%
	Projection 2	1.342	34.2%
	Projection 3	1.202	20.2%
Mean		1.287	28.7%
7.5 hr., 20 W cm^{-2} Reconstructions	Projection 1	1.287	28.7%
